# Neo-Liberalism, Policy Incoherence and Discourse Coalitions Influencing Non-Communicable Disease Strategy: Comment on "How Neoliberalism Is Shaping the Supply of Unhealthy Commodities and What This Means for NCD Prevention"

**DOI:** 10.15171/ijhpm.2019.95

**Published:** 2019-10-28

**Authors:** Samantha Battams

**Affiliations:** Southgate Institute for Health, Society and Equity, Flinders University, Adelaide, SA, Australia.

**Keywords:** Neoliberalism, Non-communicable Disease, Policy, Discourse

## Abstract

Lencucha and Thow have highlighted the way in which neo-liberalism is enshrined within institutional mechanisms and conditions the policy environment to shape public policy on non-communicable diseases (NCDs). They critique the strong (but important) focus of public health policy research on corporate interests and influence over NCD policy, and point toward neo-liberal policy paradigms shaping the relationship between the state, market and society as an area for critique and further exploration. They also importantly underline the way in which the neo-liberal policy paradigm shapes the supply of unhealthy goods and argue that health advocates have not engaged enough with supply side issues in critiques of policy debates on NCDs. This is an important consideration especially in the Asia-Pacific where trade and agricultural policies have markedly shaped production and what is being produced within countries. In this commentary, I reflect upon how neoliberalism shapes intersectoral action across trade, development and health within and across institutions. I also consider scope for international civil society to engage in advocacy on NCDs, especially where elusive ‘discourse coalitions’ influenced by neoliberalism may exist, rather than coordinated ‘advocacy coalitions.’

Underlining the importance of neoliberalism and its impact on institutions^1^ is consistent with our previous research undertaken in Australia, Malaysia and Switzerland on the links between trade, development and health sectors and implementation of global non-communicable disease (NCD) strategy.^2-6^ The influence of neoliberalism on development has previously been highlighted by Rosser, with the argument that neoliberalism provides the ideological framework for Australia’s development objectives irrespective of the government’s political persuasion.^7^ We also underscored the way in which trade and development policy are influenced by neo-liberalism, resulting in trade and economic development goals prevailing over health goals linked to NCDs, at both an international and national level.^3,5,6^ Economic development was a primary focus for development and trade sectors within and across institutions. Neo-liberal imperatives in trade and development created different ‘world views’ than those of public health practitioners and advocates. There was different ‘issues framing,’ with few opportunities to negotiate and shift ‘policy framing’^8^ due to poor intersection between health, trade and development. Along with this were different discourses and languages, including legalistic terms in trade agreements, which public health sector workers required a grasp of in order to engage in dialogue, and challenge power asymmetries in the policy environment. International health goals and policy decisions were subject to the rules of trade, however conversely health treaties are not enforceable. We drew attention to the relationship between the ‘deep core’ of neo-liberal values and assumptions, neo-liberal ideas and powerful networks linked to institutions to understand the policy environment influencing NCD strategy.^6^ Better dialogue between health, trade and development sectors and actors is required to address competing policy frames which shape the NCD policy environment, for example the perception in the trade sector that there is a ‘demand’ for unhealthy food and commodities.^4,6^

Lencucha and Thow set their focus on the neo-liberal policy paradigm against the predominant focus of public health researchers upon commercial interests within policy processes. We suggested that there were powerful alliances linked to geo-political interests and institutions for international governance across trade and health, not simply networks based upon private commercial interests,^4-6^ providing some evidence for advocacy coalitions.^9^ We called for future research that considers the various interests within policy networks aligned to NCD risk factors.^6^ We also argued the need for civil society mobilisation on issues related to trade and NCDs, with international civil society networks helping to support progress on NCDs at a national level.^2,4-6^

How can we get civil society better engaged and mobilised? Schrecker has called for consideration of the ways in which both so called ‘external factors’ (eg, globalisation) and ‘internal factors’ (eg, linked to political constituents support or otherwise for redistribution policies) within a country pose obstacles to addressing health inequalities.^10^ An obstacle to political commitment on trade/health and NCD issues may be the perception that political constituents at a national level do not know or care about complex issues around trade and health. They may be unaware of trade/health issues and their relationship to personal or familial experiences of NCDs. There has been scant media on trade/health issues (at least in Australia) until fairly recently – and so translation on trade/health issues through the media and public discourse has been limited. The advocacy that does occur (at least in Australia) appears to be largely done by political elites (eg, a small group across bureaucratic, intellectual, media and civil society circles). However, trade and health issues have been successfully politicised in other countries, including in the United States and European countries (and especially with US and European based advocacy groups working at an international level). In Australia and other countries, there is a need for greater mobilisation of civil society advocating on NCDs through engagement with the media, with the support of international civil society. There is much scope for highlighting the growing inequities in health linked to NCDs (within and across countries); bridging the link between personal and population group experiences of NCDs, commercial determinants of health (trade/health issues) and health inequities; and trade/development policies and their relationship to health and social protections to address NCDs.

However, social and critical constructionist policy theorists have criticised a focus in policy research upon who is advocating on policy, who is at the policy table and who gets their interests heard and progressed through policy processes, rather than the ‘discourses’ surrounding policy debates and who is not at the policy table,^11^ and/or the social construction (or distortion) of ‘facts’ and evidence, along with the social-political acceptability of policy proposals.^12^ Neoliberal discourse on NCDs may influence either discourse coalitions^12^ and/or advocacy coalitions,^9^ leading to the social-political acceptability of policy proposals on health at a national level.^4,5^

Challenging neoliberal discourses on NCDs (that health is primarily related to individual ‘choices’ and behaviours and unrelated to globalisation, supply issues, global marketing or inequities) within policy networks and debates should thus be a focus. It should be noted that neoliberal discourses are not just embodied in places, groups or networks associated with business interests (eg, trade/economic departments of government). Following Foucault, the power of neo-liberal discourse is in its diffusion through all parts of society, even affecting individuals’ thinking and behaviour. However, with this diffusion comes a diffuseness that can be challenged and modified.

There is scope for examination of the relationship between neoliberal discourse and paradigms and social and cultural factors, and the social-political acceptability of NCD policy proposals at a country level. A consideration is the way that both business and consumer advocacy/demands are constructed within a neo-liberal paradigm and legitimised through local political processes (involving the media). For example, in policy research conducted in Malaysia, when asking a civil servant about progress on global NCD strategy, it was discussed how Malaysia has strong imperatives for economic development and a goal of becoming a high income country by 2020, but that public health measures were sometimes seen to be add odds with this.^4^ It was protests by businesses and concerns about economic factors (eg, associated with banning smoking within restaurants or limiting the opening hours of restaurants)^4^ that appeared most legitimised in consultation processes as they presided in the media and influenced the political process (consultations) associated with the suggested introduction of NCD policy proposals. However, the neo-liberal paradigm was not only seen to be shaping consumer demand for unhealthy products^4^ but also perceptions about embedded culture; it was suggested that business (aligned with goals of economic development, but also government as a whole) did not want proposed changes (eg, tobacco bans, limited hours of restaurants, regulations on sugary drinks), and neither did some community groups (eg, as they wanted to spend after hours within restaurants, or did not want to see the ban on sugary drinks, as it was part of the culture), although these business and consumer groups were not ‘working together.’

Consistent with a social constructionist approach to policy, social and political realities were at the forefront of policy decision-making, not just technical evidence.^12^ However, as interest groups were not necessarily coordinated, it could be said that there were ‘discourse coalitions,’^12^ or groups and individuals interpreting potential courses of action and their effect through a neoliberal lens. Much research on the policy environment shaping NCDs appears to be focused upon coordinated action and networks at an international or country level, based upon commercial interests. However, discourse coalitions are not necessarily active or involved in coordinated activity within policy environments.^12^ Both commercial and consumer interests may be influenced by neoliberal ideologies within ‘discourse coalitions.’ When it comes to progress on NCDs, there is more scope to consider ‘discourse coalitions,’ upon the policy environment and challenging underlying neoliberal assumptions of such coalitions.

Conversely, and consistent with the neoliberal paradigm, influencing or creating ‘consumer demand’ for healthy products and incentivising industry through front of pack labels is perceived as one way to further both public health and business/economic goals (a passive instrument of government).^5^ Of interest will be the impact of governments working within the neoliberal paradigm to influence consumer demand, industry incentives, or supply side issues to improve health. Challenging economic rationality and the fact that economic goals prevail over social goals,^1^ or trade and development goals prevail over public health goals,^2-6^ is not only required, but also perceptions about consumer demands and rights and understanding of the socio-cultural context, along with civil society views on what influences health. Challenging neo-liberal assumptions associated with ‘discourse coalitions,’ including the way evidence is used (and misused), will be central to advocacy and progress on NCDs.

## Ethical issues

Not applicable.

## Competing interests

Author declares that she has no competing interests.

## Author’s contribution

SB is the single author of the paper.

## References

[1] Lencucha R, Thow AM (2019). How Neoliberalism Is Shaping the Supply of Unhealthy Commodities and What This Means for NCD Prevention. Int J Health Policy Manag.

[2] Battams S. Australia’s role in health governance and diplomacy in the Asia-Pacific region: Focus on NCDs. 3rd Population Health Congress (PHAA, AEA, AHPA, AFPHM); Hobart, Australia; September 2015.

[3] Battams S. The impact of neoliberalism on Australia’s rollout of global non-communicable disease strategy nationally and health diplomacy role for the Asia-Pacific region. The Australian Sociological Association conference – Neoliberalism and Contemporary Challenges for the Asia-Pacific; Cairns; November 2015.

[4] Battams S. Coordinated action across trade and health policy sectors to progress Non-Communicable Disease (NCD) strategy. PHAA 44th Annual Conference & 20th Chronic Diseases Network Conference; Alice Springs, NT; September 2016.

[5] Battams S. Challenges for Australia’s role in effectively implementing global non-communicable disease (NCD) strategy. Adelaide, SA: Southgate Institute for Health, Society and Equity, Flinders University; 2017.

[6] Battams S, Townsend B (2019). Power asymmetries, policy incoherence and noncommunicable disease control - a qualitative study of policy actor views. Crit Public Health.

[7] Rosser A (2008). Neo-liberalism and the politics of Australian aid policy-making. Aust J Int Aff.

[8] van Hulst M, Yanow D (2016). From policy “frames” to “framing” theorizing a more dynamic, political approach. Am Rev Public Adm.

[9] Sabatier P, Jenkins-Smith H. The advocacy coalition framework. In: Sabatier P, ed. Theories of the Policy Process. Colorado, USA: Westview Press; 1999:117-166.

[10] Schrecker T (2016). Globalization, austerity and health equity politics: taming the inequality machine, and why it matters. Criti Public Health.

[11] Bacchi C. Analysing Policy: What’s the problem represented to be? Frenchs Forest, NSW: Pearson Education; 2009.

[12] Fischer F. Reframing Public Policy: Discursive Politics and Deliberative Practices. Oxford: Oxford University Press; 2003.

